# Hydrogel Microvalves as Control Elements for Parallelized Enzymatic Cascade Reactions in Microfluidics

**DOI:** 10.3390/mi11020167

**Published:** 2020-02-05

**Authors:** Franziska Obst, Anthony Beck, Chayan Bishayee, Philipp J. Mehner, Andreas Richter, Brigitte Voit, Dietmar Appelhans

**Affiliations:** 1 Leibniz-Institut für Polymerforschung Dresden e.V., Hohe Straße 6, 01069 Dresden, Germany; obst@ipfdd.de (F.O.); c.bishayee@ifw-dresden.de (C.B.); voit@ipfdd.de (B.V.); 2 Organische Chemie der Polymere, Technische Universität Dresden, 01062 Dresden, Germany; 3 Institut für Halbleiter- und Mikrosystemtechnik, Technische Universität Dresden, 01187 Dresden, Germany; Anthony.Beck@tu-dresden.de (A.B.); philipp_jan.mehner@tu-dresden.de (P.J.M.); andreas.richter7@tu-dresden.de (A.R.)

**Keywords:** thermoresponsive, hydrogel, valves, poly(*N*-isopropylacrylamide) (PNiPAAm), polydimethylsiloxane (PDMS)-on-glass, microfluidics, enzyme, parallelization

## Abstract

Compartmentalized microfluidic devices with immobilized catalysts are a valuable tool for overcoming the incompatibility challenge in (bio) catalytic cascade reactions and high-throughput screening of multiple reaction parameters. To achieve flow control in microfluidics, stimuli-responsive hydrogel microvalves were previously introduced. However, an application of this valve concept for the control of multistep reactions was not yet shown. To fill this gap, we show the integration of thermoresponsive poly(*N*-isopropylacrylamide) (PNiPAAm) microvalves (diameter: 500 and 600 µm) into PDMS-on-glass microfluidic devices for the control of parallelized enzyme-catalyzed cascade reactions. As a proof-of-principle, the biocatalysts glucose oxidase (GOx), horseradish peroxidase (HRP) and myoglobin (Myo) were immobilized in photopatterned hydrogel dot arrays (diameter of the dots: 350 µm, amount of enzymes: 0.13–2.3 µg) within three compartments of the device. Switching of the microvalves was achieved within 4 to 6 s and thereby the fluid pathway of the enzyme substrate solution (5 mmol/L) in the device was determined. Consequently, either the enzyme cascade reaction GOx-HRP or GOx-Myo was performed and continuously quantified by ultraviolet-visible (UV-Vis) spectroscopy. The functionality of the microvalves was shown in four hourly switching cycles and visualized by the path-dependent substrate conversion.

## 1. Introduction

Stimulated by advancements in the fabrication of miniaturized microfluidic devices and the miniaturization of biochemical analysis, microfluidic systems have evolved to a powerful method in biomedical and chemical research over the last decades [1,2]. As these “labs-on-chips” allow a high integration density of multiple functions such as mixing and separation of small amounts of fluids, even complex samples can be prepared, processed, and analyzed in a short time. Consequently, lab-on-chip devices have a high potential for biomedical research e.g., to perform immunological assays [3,4] to cultivate cells [5] and to construct organs-on-a-chip for toxicological studies and disease modeling as well as personalized medicine [1,2,6,7]. Moreover, miniaturized flow-through devices offer high potential for the (bio) catalytic investigations as they are predestinated for the rapid screening of several reaction parameters and/or the realization of complex cascade reactions with a low material consumption and continuous product supply. Consequently, several biocatalytic conversions were integrated into microfluidic devices e.g., to screen enzymatic activities [3,8,9] or to perform continuous catalytic reactions [10,11,12,13,14,15,16,17]. Thereby, enzymes were either dissolved in an appropriate fluid and flushed through the device or immobilized therein. Especially for establishing multi-step cascade reactions with incompatible reaction steps, the immobilization of biocatalysts in compartmentalized flow-through devices is an elegant method and the construction of such devices was demonstrated by several research groups [11,18], including ours [15]. Within this approach, the substrate solution is pumped through the device and stepwise converted upon contact with the respective immobilized enzymes, thereby avoiding cross-inhibitions and enabling optimization of the reaction conditions for the individual steps. Thus, frequently arising problems from biocatalytic cascade reactions can be solved [19,20]. However, despite the suitability of microfluidic devices for performing parallelized tasks, so far only serial and no parallel multi-step reactions are described. Consequently, the potential of performing multiple types of cascade reactions and achieving rapid screening of multiple reaction parameters is not fully utilized yet. Moreover, in previous examples, no flow control elements were applied to adjust the filling and flushing of the individual compartments and thereby truly to achieve large scale integration. For instance, such a flow control could allow determining which processes should take place on the chip at a certain time and facilitate the experimental processes e.g., by easier variation of the residence time of the fluid in the compartments.

**Figure 1:**
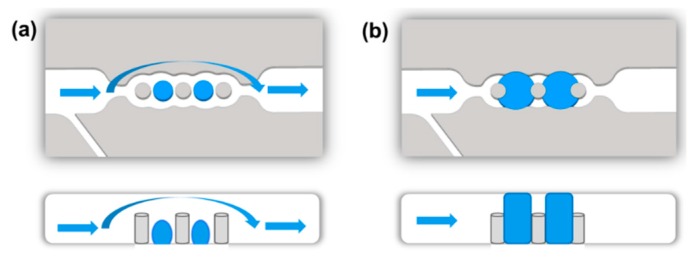
Concept of a hydrogel microvalve in a microfluidic channel in top view (**top**) and side view (**bottom**) with blue arrows symbolizing the fluid flow. (**a**) In the shrunken state, the hydrogel does not block the microfluidic channel and the valve is opened. (**b**) The swollen hydrogel fully blocks the microfluidic channel and impedes the fluid flow. Thus, the valve is closed.

Stimulated by this, we aim to integrate microvalves as fluid control elements into compartmentalized microfluidic devices with immobilized biocatalysts and showcase the long-time functionality in a multi-step reaction. For this purpose, we utilize the previously established concept of hydrogel microvalves which controllably block or open the microfluidic channel. We chose to apply microfluidic devices formed by assembling photostructured polydimethylsiloxane (PDMS) sheets on a glass support (PDMS-on-glass devices). Such devices are valuable tools in research as they can be rapidly prototyped and due to their transparency the fluid flow can be easily visually observed. Additionally, the immobilization of biocatalysts in such devices by several methods such as binding to the glass surface [11] or to other support materials [10] as well as entrapment into a photopatterned matrix [14,21] was previously achieved. In addition, the integration of microvalves in PDMS-on-glass microfluidic devices and switching by a great variety of stimuli was focus of extensive research and multiple concepts were realized [22,23,24]. Valves in single layer microfluidic devices can be realized by intrinsically active materials such as hydrogels. Hydrogels are crosslinked polymer networks that swell in water and change their swelling degree in response to certain stimuli [25]. Thus, controlled change of the swelling degree of a hydrogel placed in a microfluidic channel allows determining whether the fluid can or cannot pass the hydrogel, i.e., whether the valve is opened or closed. To define the position of the hydrogel and prevent movement under a fluid flow it is hold in place by valve seats (Figure 1).

For integration of hydrogel valves into the microfluidic devices, two strategies are described. In one of these approaches, hydrogels are microstructured by photopolymerization on a glass slide and a PDMS sheet is aligned on top. With this technique, high numbers of hydrogel valves can be simultaneously integrated into one microfluidic chip [26]. Alternatively, the microstructured hydrogels are manually integrated into the PDMS sheet by the pick-and-place method prior to the assembly of the PDMS-on-glass device. Depending on the chemical composition of the hydrogels, the switching of the valves was achieved by stimuli such as the temperature [27], light [28,29,30], pH value [27,31], electric [32] or magnetic [33] fields, and chemicals e.g., glucose [34] or ethanol [35]. Hydrogels based on PNiPAAm are frequently applied as thermoresponsive microvalves as PNIPAAm possesses a LCST of 33 °C and thus shrinking of the hydrogel and can be triggered by moderate heating [36]. With micrometer-sized hydrogels, response times in the range of a few seconds were achieved [26,37]. In proof-of-principle studies, the valve characteristics such as response time, pressure resistance, and cyclic stability were widely studied and related to the composition and size of the hydrogel and the general valve design [27,38,39,40,41]. The value of the valve concept was subsequently impressively demonstrated by establishing the DNA amplification by the polymerase-chain-reaction (PCR) on a valve-controlled microfluidic chip [42,43,44]. However, as stated above further fields of application of the microvalves e.g., for the establishment of multi-step cascade reactions on microfluidic devices were not yet opened up.

**Figure 2:**
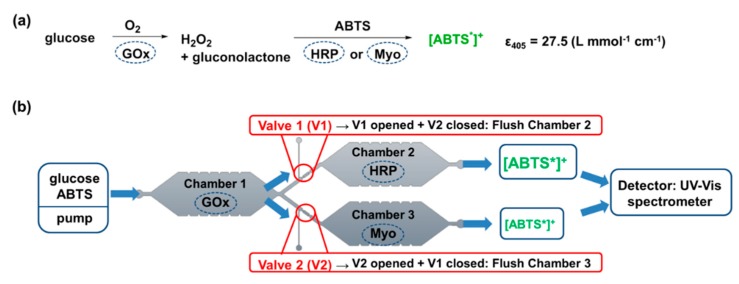
(**a**) Chemical reaction for the two-step enzyme-catalyzed conversion of glucose and ABTS to the UV-Vis active [ABTS*]^+^. (**b**) Integration of the enzyme-catalyzed reactions in a microfluidic device with three separated reaction compartments and immobilized enzymes. Switching between the bi-enzymatic reactions GOx-HRP and GOx-Myo is done by hydrogel valves and the concentration of the formed [ABTS*]^+^ is measured by UV-Vis spectroscopy.

To fill this gap and visualize the compatibility of the microvalves with biocatalytic reactions, we developed a PDMS-on-glass microfluidic device with three separated reaction chambers whereby two of them are arranged in parallel (Figure 1). Thermally controllable microvalves based on PNiPAAm hydrogels were applied to either open or close the parallel chambers and thereby to guide the fluid through the desired compartment. As a model system, the enzymes glucose oxidase (GOx) and horseradish peroxidase (HRP) as well as the protein myoglobin (Myo) were immobilized in a hydrogel matrix in the reaction chambers. Thereby, the two bi-enzymatic cascades GOx-HRP and GOx-Myo were formed which both can stepwise convert the substrates glucose and 2,2′-azino-bis(3-ethylbenzothiazoline-6-sulfonic acid) diammonium salt (ABTS) to finally yield the UV-Vis active radical cation [ABTS*]^+^ (Figure 2a) [45,46]. Depending on the opening or closing of the mico-valves in front of the parallel chambers, either the reaction GOx-HRP or GOx-Myo is performed in the microfluidic device. As the specific activity of Myo is lower than that of HRP, less [ABTS*]^+^ is formed by this reaction path. Consequently, the model allows visualizing the opening and closing functionality of the valves by UV-spectroscopy (Figure 2b).

Here, we present the design and fabrication of the PDMS-on-glass microfluidic device with integrated hydrogel microvalves as well as the basic design criteria. Moreover, the method for the immobilization of spatially separated enzymes in these devices is outlined and the enzymatic assay for determining the catalytic activity is detailed. Finally, the results of performing the parallelized enzymatic reactions under a continuous flow with multiple switching cycles of the valves are shown. In this respect, handling issues of the device are discussed as well.

## 2. Materials and Methods

### 2.1. Chemicals and Materials

*N*-isopropylacrylamide (NiPAAm), *N*,*N*′-methylene-bis-acrylamide (BIS), poly(ethylene glycol) diacrylate (PEGDA, Mw 700 g/mol), 2-(dimethylamino)ethyl methacrylate (DMAEMA, 98%), 2-hydroxyethyl methacrylate (HEMA, ≥99%), lithium phenyl-2,4,6-trimethyl-benzoylphosphinate (LAP) 3-(trichloro-silyl)propyl methacrylate (TPM, ≥90%), 2,4,6-trimethylbenzoyl chloride (97%), peroxidase from horseradish (HRP, essentially salt-free, lyophilized powder), glucose oxidase from *aspergillus niger* (GOx, lyophilized powder), myoglobin from equine skeletal muscle (Myo, essentially salt-free, lyophilized powder), and 2,2′-azino-bis(3-ethylbenzothiazoline-6-sulfonic acid) diammonium salt (ABTS, ≥98%) were purchased from Sigma-Aldrich (St. Louis, MO, USA). D-(+)-glucose (anhydrous) was obtained from Fluka Bio Chemika (Muskegon, MI, USA). Ammonium hydroxide 28–30 wt %) was obtained from Acros Chemicals (Trenton, NJ, USA). Ethanol and isopropyl alcohol (IPA) were purchased from Fisher Chemicals (Pittsburgh, PA, USA). Hydrogen peroxide (H_2_O_2_, 35%) and the glass slides (Menzelglas, Thermo Scientific, 26 × 76 × 1 mm^3^) were purchased from Merck (Braunschweig, Germany). Polydimethylsiloxane (PDMS, Sylgard 184) was purchased from Dow Corning (Midland, MI, USA). Both HEMA and DMAEMA were passed through a column filled with neutral aluminum oxide to remove the stabilizers prior to use. All other chemicals were used as received. Filtered and deionized water (MilliQ) was obtained by Milli-Q^®^ Gradient A10^®^ from Merck Millipore (Burlington, MA, USA). Poly(oxymethylene) (POM) and poly(methyl methacrylate) (PMMA) were purchased from PCH Technischer Handel GmbH (Dresden, Germany). Water-soluble core/shell quantum dots (QD) from AgInS/ZnS were synthesized as previously reported [15,47] and kindly provided by Oleksandr Stroyuk (TU Dresden, Institute for Physical Chemistry, Dresden, Germany).

### 2.2. Measurement of Enzyme Activities

The catalytic activities of GOx and HRP were determined in an ABTS assay in PBS buffer (100 mmol/L, pH 7.4) at 25 °C according to a previously described procedure [15]. The catalytic activity of Myo was determined with 5 mmol/L ABTS and 5 mmol/L H_2_O_2_. Within the experimental setup, this concentration of H_2_O_2_ resulted in maximum activity of the Myo. Enzyme inhibition was noted at a H_2_O_2_ concentration above 50 mmol/L. The formation of [ABTS*]^+^ from ABTS was monitored by UV-Vis spectroscopy (SPECORD 210 PLUS, Analytik Jena AG, Jena, Germany) in single-use cuvettes at a wavelength of 405 nm for 10 min. All measurements were carried out in triplicates. The catalytic activities were calculated from the slopes of the time-dependent absorption using Lambert-Beers law. The extinction coefficient ε_405_ of (ABTS*)^+^ was determined experimentally by a calibration (ε_405_ = 27.5 L/(mmol cm) [15]. The following results were obtained at pH 7: HRP: 76.2 U/mg, GOx: 14.3 U/mg, Myo: 0.33 U/mg. As the data show, Myo has the lowest specific activity of the catalysts. However, at pH 6.0 the activity of Myo is higher than at pH 7.4 (factor 2.0) so that pH 6.0 was applied for the microfluidic experiments.

The enzyme-catalyzed conversion in microfluidic devices depends on several parameters such as the flow rate and correspondingly the residence time as well as the substrate concentration. These relations were subject of our previously published investigations [14,15]. Moreover, apart from the catalytic activity of the free enzymes, the activity of immobilized enzymes is crucial to evaluate the quality of the immobilization technique and the performance of the flow-through device. Consequently, respective calculations were also previously presented [15].

### 2.3. Enzyme Immobilisation in Photopatterned Hydrogel-Enzyme-Dots

**Figure 3:**
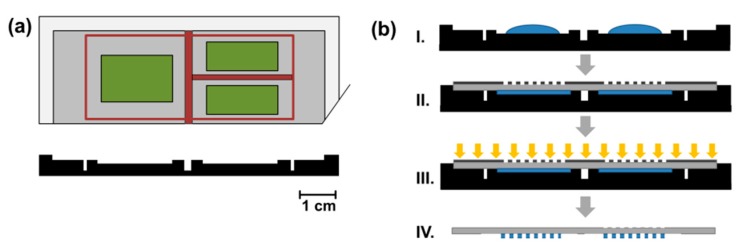
Production of spatially separated hydrogel dot arrays with a POM mould. (**a**) Scheme of the POM mould in the top view and side view. Colors indicate the three-dimensional structure of the mould. Green: chambers which are filled with the hydrogel precursor solution (depth: 100 µm), red: channels which serve as a drain for excess hydrogel precursor solution and prevent intermixing (depth: 3 mm), grey: surface where the glass slide is placed, white: boundary for the alignment of the glass slide (height: 1 mm). (**b**) Scheme of the polymerization process. Filling of the hydrogel precursor into the mould (I), alignment of glass slide and photomask (II), UV-irradiation (III), hydrogel dots on glass slide (IV).

The hydrogel precursor solution for the immobilization of enzymes was prepared as previously reported [14,15]. In brief, 1000 mg (1.43 mmol) PEGDA, 101.4 µL (0.66 mmol) DMAEMA, 43.4 µL (0.40 mmol) HEMA and 39.9 mg (0.14 mmol) LAP were dissolved in 1.727 mL MilliQ water and stirred in the dark until LAP was completely dissolved. Subsequently, the three different lyophilized proteins were separately dissolved in the hydrogel precursor solution and the following enzyme concentrations were adjusted: GOx: 1.17 mg/mL, HRP: 0.40 mg/mL or 0.13 mg/mL, Myo: 2.30 mg/mL. These concentrations were chosen on the basis of initial experiments as they result in reliable enzyme immobilization and allow direct quantification by UV-Vis sepectroscopy under flow-through conditions [15]. Glass slides were pretreated by RCA cleaning and subsequent functionalization with TPM under reduced pressure according to a standard procedure [15]. For photopolymerization of the hydrogel and spatially controlled enzyme immobilization, the enzyme-containing hydrogel precursor solutions were pipetted into a mould made from black POM featuring three separate 100 µm deep cavities according to a previously published procedure [15]. To prevent intermixing of the hydrogel precursors i.e., to precisely control the composition of the hydrogel arrays, the cavities are separated from one another by open channels (Figure 3a). The POM mould was produced in-house by mill-cutting on a four-axis CNC milling machine (DMU 50, DMG MORI, Bielefeld, Germany). The three different hydrogel precursor solutions containing either GOx, HRP, or Myo (60 µL) were filled into the chambers of the mould and a TPM-functionalized glass slide was aligned on top such that no intermixing of the hydrogel precursors occurred. Subsequently, a polymer photomask featuring transparent dots with a diameter of 350 µm was placed on top of the glass slide. The photopolymerization was performed with a UV lamp (DELOLUX 04, DELO, Windach, Germany, optical power on the sample surface: 8 mW/cm^2^, emission spectrum: 315–500 nm, irradiation time: 7.5 s) in an air-conditioned lab (22 °C). Hydrogel dots with a defined height (as predefined by the mould) and diameter (as predefined by the photomask feature size) covalently attached to the glass slide were obtained. Afterwards, the glass slides were separated from the mould, rinsed with MilliQ to remove the unreacted hydrogel precursor solution, and stored in PBS buffer (100 mM PBS, pH 7.4) at 4 °C overnight to completely remove loosely attached hydrogel material and enzymes (Figure 3b). To experimentally confirm that no intermixing of the hydrogel precursors occurs prior to the photopolymerization, they were stained with water-soluble core/shell quantum dots from AgInS/ZnS with different emission colors according to a previously reported method [15]. With this approach, intermixing of the hydrogel precursors can be visualized by variations of the emission colors from the respective hydrogel arrays. However, as no such variations occurred, the accuracy of the process was proven (Figure S1).

### 2.4. Synthesis of Hydrogel Microvalves

Hydrogels for the hydrogel microvalves were synthesized by photopolymerization of an aqueous precursor solution. Thereby, either 1.5 or 5.0 mol % of the crosslinker BIS were applied. The hydrogel precursor solution A was produced by dissolving the monomers NiPAAm (707.3 mg, 98.5 mol %) and BIS (14.5 mg, 1.5 mol %) as well as the photoinitiator LAP (18.4 mg) in distilled water (5.0 mL). In case of hydrogel precursor B, only 95 mol % of NiPAAm and consequently 5 mol % of BIS were used. Hydrogels were formed in a moulding process similar to the process shown in Figure 2b. However, instead of the POM, a glass slide (76 × 26 × 1 mm³) with a spacer of Scotch tape (150 µm) was used as a mould. POM moulds were not as useful for this process, as the hydrogels are not covalently attached to the glass slide during the polymerization (for covalent attachment see Section 2.3) and thus detachment occurs. As the glass slides can be easier separated from one another than from the POM mould, less mechanical forces act on the hydrogels which consequently stay attached to the glass. The hydrogel precursor solution was pipetted into the spacer-defined cavity under argon atmosphere in a glovebox and covered with a thin glass slide (thickness: 0.15 mm). Hydrogels were produced with two different sizes by placing polymer photomasks with transparent dots (diameter either 500 or 600 µm, array of 10 × 10 dots) on top of the thin glass slide. Finally, the hydrogel precursor was irradiated with a UV-LED array (CBM-120 Mosaic, Luminus Inc., Sunnyvale, CA, USA, 15 s, intensity on the sample surface: 1 mW/cm^2^) in combination with an in-house developed lense system. After the polymerization, the photomask was removed and the two glass slides were separated from one another whereby the cylinder-shaped hydrogels sticked to the thin glass slide. The slide with the hydrogels was placed in a petri dish with distilled water for 24 h. To completely wash away unpolymerized hydrogel precursor solution, three washing cycles with fresh deionized water were carried out. For a picture of an array of hydrogel-valves see Figure S2.

### 2.5. Design and Production of the Heat Pad

The heat pads for the electro-thermal switching of hydrogel microvalves were produced from polyimide foils (thickness: 50 µm) coated with copper (25 µm) on both sides. Conducting paths were etched into one side using a standard printed circuit board (PCB) process. In short, the copper surface was coated with a photoresist which was subsequently UV-structured and developed. Then, the uncovered copper was etched and the resist was removed. Subsequently, two resistors (30 Ω, one-component carbon conductive varnish SD 2843 HAL, PETERS Speziallacke für die Elektronik, Kempen, Germany) were placed on the contact zones by screen print (Figure S3). The resistors are used as heating elements to control the polymer actuators in the valve seat. The size and relative position of the two resistors are adjusted to the position of the valve structures in the microfluidic design. Heating of the resistors was done by applying a voltage of 4 V with a power supply (QUAT Power, LN 3003, Pollin Electronic, Pförring, Germany). A switch was integrated to control the current supply and consequently the heating of each resistor. To determine the voltage required for the heat-induced shrinking of the microvalves, the relation between voltage and resulting temperature was measured. For this purpose, the heat pad was placed in the aluminum holder (see Section 2.6) and a glass slide (same type as used for the microfluidic chips) was placed on top. Subsequently, the voltage was turned on and the temperature of the glass surface above the resistor was measured with a temperature sensor. At a voltage of 4 V, the temperature of the heating resistor reached 40 °C, which is sufficient to induce shrinking of the hydrogel. However, to account for cooling effects upon a continuous fluid flow in the microfluidic device, a higher voltage (5 V) was applied to reach a higher temperature (48 °C). With this setup, both microvalves could be opened independently by heating of the respective heat resistor as only slight passive warming (up to 28 °C) of the other resistor occurred. The rapid dissipation of the heat is promoted by the high thermal conductivity of the aluminum holder on which the heat pad and the microfluidic chip are placed.

### 2.6. Production and Assembly of Microfluidic Devices

Photomasks for the microfluidic devices were designed with the CAD software Autodesk Inventor and produced by photo plotting (MIVA 26100 ReSolution, MIVA Technologies, Schönaich, Germany) on black-and-white flat films. PDMS sheets were fabricated by hard and soft lithography in a standard process [48]. In a first step, glass substrates were rinsed with acetone, IPA, and MilliQ and baked at 150 °C for 20 min. Three resist layers (DFR, 50 µm) were stepwise laminated on the glass slide and each heated to 85 °C for 3 min. Afterwards, the resist was exposed to UV light through a polymer photomask for 90 s followed by a post-exposure heating for 40 min at 85 °C. Then, the resist was developed, rinsed, and finally heated to 85 °C for 1 h. PDMS sheets were produced by pouring a mixture of elastomer base and curing agent (mass ratio 10:1, following the instructions of the supplier) over the master, degassing, and curing for 3 h at 60 °C. Subsequently, the PDMS sheets were peeled off from the master and perforated with a biopsy punch (kai industries co., ltd, Ø: 1.5 mm) to form the fluidic connections.

To integrate the microvalves in the microfluidic device, the wet hydrogels (see Section 2.4) were first placed on a piece of adhesive tape and dried in an oven (40 °C, 10 min). As the dry hydrogels stick much less to the smooth surface of the tape than to a glass surface, this approach facilitated the manual handling. Subsequently, the hydrogel pieces were lifted from the tape with a cannula and placed in the valve seats of the PDMS sheet (pick-and-place method) according to a previously described process [49]. Thereby, the PDMS was observed through a microscope (10-fold magnification) to ensure accurate placement of the hydrogels. Glass slides with conditioned enzyme-loaded hydrogel dots were rinsed with MilliQ. Subsequently, cotton swabs were used to first remove the MilliQ from the glass surface surrounding the hydrogel array and then clean this area with IPA. In this process, the hydrogel arrays were not touched with the cotton swabs. For the investigation of the fluid flow and functionality test of the valves, native glass slides (without hydrogel-enzyme dots) were rinsed with IPA and water and dried in a nitrogen stream. Microfluidic devices were formed by assembling the PDMS sheets on the glass slides which were then covered with an aluminium frame with a transparent window of PMMA (Figure S4), aligned on the heat pad, and clamped in an aluminium holder (Figure S5). To connect the microfluidic devices with the flux pump (Landgraf syringe pump, LA 30, Landgraf Laborsysteme, Langenhagen, Germany), cannulas were used which were on the one end inserted into PTFE tubes and on the other into the punched inlet of the PDMS sheet. Subsequently, the fluids were pumped into the device with a flow rate of 5, 10, or 20 µL/min. For visualization of the fluid flow, Milli-Q stained with blue ink (5 vol %) was used. To check the functionality of the valves, both Milli-Q and PBS buffer (1 mmol/L or 100 mmol/L, pH 6.0 or 7.4) were applied. Prior to use, the fluids were degassed in a Schlenk flask by performing 5 freeze-thaw-cycles using argon as inert gas and liquid nitrogen for freezing. Microfluidic devices were first flushed with the fluids with the heating pad turned off to achieve swelling (closing) of both hydrogel microvalves. Subsequently, one of the valves was shrunken by heating of the corresponding resistor. The functionality of each type of the microvalves (hydrogel A or B with either 500 or 600 µm) was tested on three independent microfluidic devices. Every 5 min the valves were switched i.e., the formerly opened valve was closed and the formerly closed valve was opened. For conditioning [50], this procedure was repeated four times. Subsequently, cyclic stability of the microvalves was tested by performing five opening-closing cycles, both with a short cycle (5 min) and a long cycle time (1 h).

### 2.7. Measurement of Enzymatic Activity in Microfluidic Devices

To measure the enzymatic activity in the device, a substrate solution (glucose and ABTS, 5 mmol/L) in PBS buffer (100 mmol/L, pH 6.0) was pumped through the microfluidic device at a flow rate of 5 or 10 µL/min. The choice of substrate concentration and flow rate was based on previous investigations regarding the working range of this type of microfluidic devices [14,15]. Most importantly, upon using the above outlined enzyme amount, these conditions allow direct quantification of the conversion by UV-spectroscopy without the need of dilution or concentration of the samples. For spectroscopic analysis i.e., the quantification of the formed [ABTS*]^+^, the two outlets of the device were unified with a T-shaped PTFE tube which was connected to a flow cuvette (volume: 50 µL, path length: 1.5 mm, Helma GmbH, Müllheim, Germany). The cuvette was placed in a UV-Vis spectrometer (Cary 50, Varian Inc., Palo Alto, CA, USA). The absorption of the product solution flowing through the cuvette was continuously measured every 10 s at a wavelength of 405 nm. Due to the offline measurement and the time required for filling of the outlet tube of the microfluidic chip and cuvette with the product solution, the enzymatic activity within the microfluidic device was measured with a timely delay (15 min in case of a flow rate of 10 µL/min). Consequently, after switching of the valves and changing the fluid pathway between the reactions GOx-HRP and GOx-Myo, the change of the measured absorbance occurred after this delay time. The time between opening and closing of the valves was therefore set to 1 h to reach a constant absorbance i.e., a plateau of the time-dependent absorbance. The reproducibility of the enzymatic conversion in the microfluidic devices was previously shown with the enzyme cascade GOx and HRP by performing flow-through measurements (3 h) on at least three independent microfluidic devices [15]. The same experiments were carried out with the enzyme cascade GOx and Myo (Supplementary Materials—Chapter 6). To visualize the long-time functionality of the microvalves, three independent devices with immobilized enzymes and integrated microvalves were produced and analyzed under flow-through conditions (four opening-closing cycles of the microvalves, durance of the experiments 4 to 7 h)

## 3. Results and Discussion

### 3.1. Microfluidic Device with Parallel Compartments

**Figure 4:**
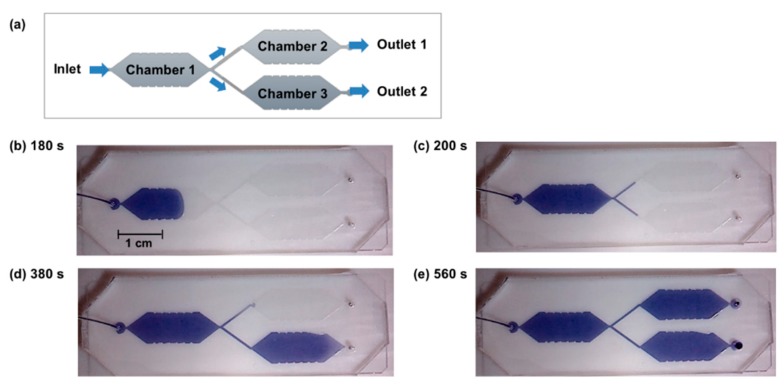
Investigation of the filling of a microfluidic device with three separated reaction chambers and a Y-junction without microvalves. The design is schematically shown in (**a**). An aqueous solution of blue ink is used for visualization and pumped at a flow rate of 5 µL/min. The pictures (**b**–**e**) are taken at different times and visualize that no parallel flushing of the rear chambers occurs.

The purpose of the herein presented study is to show a proof-of-concept for the performance of multi-step catalytic processes in microfluidic devices with serial and parallel aligned compartments. As outlined in the introduction, reproducible fluid flow and controlled filling of the individual compartments are essential. Consequently, these two aspects were investigated with the microfluidic design with three compartments (Figure 4a). Thereby, it was aimed to show, whether the microvalves are indeed required for controlled filling of the parallel reaction chambers or whether this can be achieved without integrating the valves.

The experiments regarding the filling and flushing of the device were performed with aqueous ink solution for better visualization. Thereby, flow rates of 5, 10, and 20 µL/min were applied as these are suited for performing the intended enzymatic reactions (see Section 2.7). As intended by the chamber design with a conical widening inlet, the first chamber of the device was filled without the enclosure of air bubbles (Figure 4b). At the Y-junction, the fluid equally spread into two parts (Figure 4c). However, when the fluids reached the conical widening inlets of the two parallel chambers, no parallel filling occurred. Instead, only one chamber was filled whereas the fluid flow stopped in the other channel (Figure 4d). Only when the first of the parallel chambers was completely filled and the fluid reached the outlet of the device, filling of the previously empty chamber started. Finally, the fluid flowed only out of one outlet (Figure 4e). Also with increased flow rates of 10 and 20 µL/min no parallel filling and flushing occurred.

In addition to the above described filling experiments, a different concept of the fluid actuation in the microfluidic device was applied. Specifically, instead of actively pumping the fluid into the device, a passive connection of the inlet with a fluid reservoir was established. Two pumps were connected with the two fluid outlets and were run in a pulling mode (2.5 µL/min each). Thereby, it was aimed to pull the fluid into both of the parallel chambers to obtain simultaneous filling and flushing. However, as it turned out this approach of pulling is not compatible with the PDMS-on-glass microfluidic device. Upon pulling, a negative pressure was induced in the device as filling by the fluid reservoir was not fast enough to compensate the volume withdrawn from the device. Due to the softness of the PDMS, this resulted in its bending towards the glass slide. Furthermore, because of the gas permeability of the PDMS, air bubbles were soaked into the device.

To conclude, none of these approaches allowed the controlled filling and flushing of the parallel microfluidic chambers. Consequently, with these experiments, the need for flow control elements in the microfluidic design is illustrated and hydrogel-valves were integrated in a next step.

### 3.2. Integration of Hydrogel Microvalves in the Microfluidic Device

As outlined in the introduction, hydrogel microvalves can be integrated into microfluidic devices either by direct polymerization inside the device or by the manual pick-and-place method. Especially the pick-and-place method is compatible with preformed photopatterened hydrogel-enzyme-dots on glass slides and was thus chosen within the herein presented study. The synthesis and integration of the microvalves is described in the following section.

**Figure 5:**
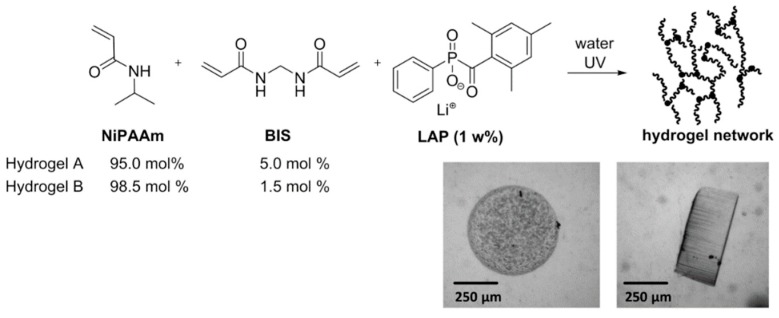
Synthesis of PNiPAAm hydrogels with BIS as a crosslinker. Microscope images show a photopolymerized cylinder of hydrogel A in the swollen state in top (**left**) and side view (**right**).

Cylindrical-shaped hydrogels based on the monomer NiPAAm and the crosslinker BIS were synthesized by photopolymerization of an aqueous hydrogel precursor solution. To obtain a selection of different hydrogels and to investigate their functionalty as valves in the microfluidic device, both the composition and the size of the hydrogels were varied. In this respect, two hydrogel compositions with either a high crosslinker content (hydrogel A, 5.0 mol % BIS) or a low crosslinker content (hydrogel B, 1.5 mol % BIS) were synthesized (Figure 5). A high crosslinker content of hydrogels usually results in lower swelling ratios and higher mechanical stability. To account for the different swelling ratios, hydrogel pieces were formed with a mask either featuring dots of 500 or 600 µm. In all cases, mechanically stable and evenly shaped uniform hydrogels were obtained (see Figure S2).

After the synthesis of the microstructured hydrogels, their functionality as microvalves in the microfluidic design was investigated. For this purpose, the hydrogels were first manually placed into the valve seat in the PDMS, microfluidic devices were assembled, and the valves were repeatedly switched. Thereby, the hydrogels were analyzed with a microscope to measure the time required for triggered swelling and shrinking and the successful switching was derived from observations of the fluid flow. Clearly, a functional valve must allow the fluid to pass when it is in the open state and fully prevent the fluid flow when it is in the closed state (for the valve concept see Figure 1).

**Figure 6:**
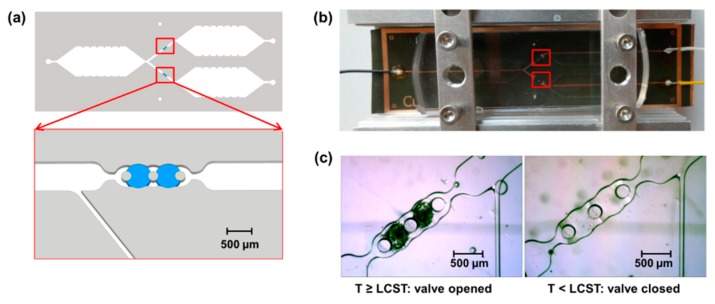
(**a**) Schematic design of the microfluidic device with three reaction chambers, two valves, and bypass channels in front of the valves. Each valve is formed by two hydrogel cylinders (blue) placed between the three in-line ordered PDMS pillars. (**b**) Photograph of a PDMS-on-glass microfluidic chip placed on a heat pad of PEI foil which is connected to a power supply and clamped in an aluminum frame. Red squares indicate the location of the valves which are placed above the two heat resistors of the heat pad. (**c**) Microscope images (10-fold magnification) of the hydrogel valves in the opened (shrunken) and closed (swollen) state. In the swollen state, the refractive index is similar to that of water and thus the contrast between hydrogel and fluid is low.

For integration of the valves into the microfluidic design, three PDMS pillars were established as support structures inside the valve seat in the microfluidic channel as they reliably hold the microvalves in place and at the same time do not hinder the fluid flow. Two pieces of hydrogel were placed in each valve to ensure reliable closing functionality (Figure 6a). Bypass channels were created in front of the valves to discharge the fluid in case of closed valves and thereby prevent destruction of the valves and/or fluid leakage upon continuous fluid pumping. Swelling and shrinking of the valves was initiated by the heat pad (Figure 6b). The functionality of the valves was both deduced from the observation of the fluid flow in the microfluidic chambers and from microscopic evaluation of the hydrogel valves (Figure 6c). As the microscope images show, the shrunken hydrogel can be readily recognized due to the high refractive index. Upon swelling, the refractive index decreases and the hydrogel becomes transparent. Thus, in the closed state of the valve, the hydrogel is hardly visible by microscopy but opening of the valve can be immediately recognized due to the rapidly increasing refractive index.

As it was recognized in the experiments, very careful handling and precise placement of the hydrogels in the PDMS was crucial to ensure sealing of the microfluidic device and achieve blocking of the channel upon swelling of the valve. Apart from that, upon heating the microfluidic device with the heat pad to shrink the hydrogels, gas bubbles were formed in the region of the valve as a side effect. Subsequently, the gas bubbles were transported downstream through the narrow channel and towards the conical widening inlet of the respective chamber. However, as the fluid could flow around the bubble in this zone, the bubbles were not transported towards the outlet. The gas bubbles disappeared when the respective valve was not heated anymore, which can be explained both by the increased solubility of the gas in the cooled fluid and by the gas permeability of the PDMS that enables release of gas from the device. Though, upon long-time heating of one valve (1 h), large gas bubbles were formed which eventually extended to the microfluidic chambers. The flow resistance in the microfluidic channels was thereby strongly increased. Consequently, it occurred that the channel was not flushed anymore although the respective valve was in the open state. As these effects clearly hinder the reproducible long-time performance of enzymatic reactions in the device, it was aimed to circumvent the formation of bubbles. For this purpose, the fluids were degassed by freeze-thaw-cycles prior to use. As a result, significantly fewer bubbles were formed in the microfluidic device upon heating. It appeared that this small amount of gas was released from the hydrogels upon the first shrinkage. This assumption was confirmed in a control experiment without integrated hydrogels as thereby no gas was formed in the device at all.

Finally, after optimization of the handling and operation process, functional microvalves which reliably opened and closed in five opening-closing cycles with a durance of both 5 min and 1 h were obtained. Thereby, no difference in the functionality occurred upon using the different hydrogel compositions A and B and the different sizes of 500 or 600 µm in the swollen state. Clearly, the swelling degrees of both the hydrogels A and B were sufficient for blocking of the microfluidic channel and thus closing of the valves. On the other hand, opening of the microvalves was reliably achieved both with small (500 µm) and big (600 µm) hydrogel particles. After switching of the heat pad, shrinkage and swelling of the hydrogels occurred in a few seconds (full opening: 4 s or less; full closing: 6 s or less). As optimization of the response time was not the main goal of this study, hydrogel A with 600 µm diameter was selected for further experiments based on handling issues and considerations of the mechanical stability. Due to the larger diameter, handling of this hydrogel and placement into the valve seat was considerably facilitated. Furthermore, the higher crosslinking degree likely results in higher pressure stability. Still, it has to be noted that very precise insertion into the PDMS was crucial to obtain functional devices and experimental expertise was thus required.

### 3.3. Parallelized Enzymatic Cascade Reactions in Microfluidic Devices

**Figure 7:**
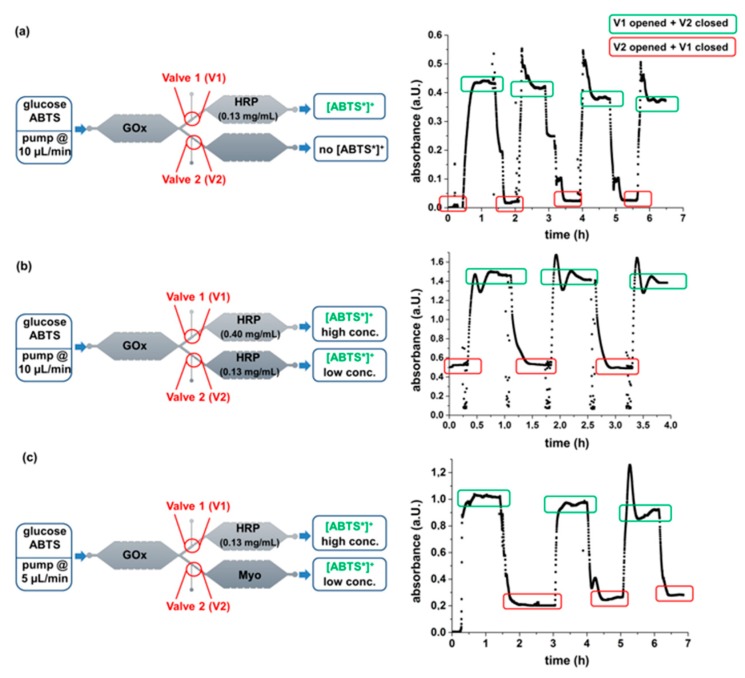
Schemes of enzymatic reaction performed in microfluidic devices with an ABTS/glucose substrate solution (5 mmol/L) (left) and results of the corresponding continuous UV-Vis measurements (right). (**a**) Microfluidic device with only GOx and HRP, (**b**) Microfluidic device with GOx and two different amounts of HRP, (**c**) Microfluidic device with GOx, HRP, and Myo. Three (**b**,**c**) or four (**a**) switching cycles of the microvalves were performed. The flow rate of only 5 µL/min in experiment (**c**) was chosen because of the low activity of Myo. The UV-Vis measurement was performed offline and thus changes of the absorbance occurred with a timely delay of 15 min with respect to the time point of the switching.

Finally, microvalves were applied in microfluidic devices with hydrogel-immobilized enzymes to control parallelized enzymatic cascade reactions. For this purpose, the biocatalysts GOx, HRP, and Myo were separately immobilized in three hydrogel arrays on a glass slide. Thereby, the size and location of the arrays was tailored to the microfluidic design with three reaction chambers to allow their integration into PDMS-on-glass microfluidic devices. With the arrangement of the hydrogel arrays, both the bi-enzymatic cascades GOx-HRP and GOx-Myo were formed. Upon pumping the glucose and ABTS containing substrate solution through the device, the substrates penetrate into the hydrogel-enzyme-dots and are catalytically converted. In a first step, glucose is oxidized to gluconolactone by GOx. In this process, H_2_O_2_ is formed. Subsequently, HRP catalyzes the conversion of ABTS to [ABTS*]^+^ whereby H_2_O_2_ is consumed. Consequently, the concentration of the formed [ABTS*]^+^ depends on the catalytic activity in the device. To visualize both the functionality and the versatility of the design, three different types of microfluidic devices were produced with varying enzyme amounts of HRP or Myo in the two parallel reaction chambers (Figure 7a–c, left). Thereby, based on the measurements of the catalytic activities (see Section 2.2) the amount of the enzymes was adjusted such that [ABTS*]^+^ was formed in clearly different concentrations on the two fluid pathways. This implies that the conversion and thus absorbance is thoroughly depending on the opening and closing of the hydrogel microvalves. Apart from that, the flow rate of the substrate solution was adjusted such that a well detectable catalytic conversion occurred. Clearly, the residence time of the substrate containing fluid within the microfluidic device and thus the reaction time for catalytic conversion decreases linearly with increasing flow rate as it was previously shown [14,15]. With the given microfluidic design and size of the hydrogel dots, the residence time of the fluid within each chamber is 60 s at a flow rate of 10 µL/min and consequently 120 s at 5 µL/min (for calculation see Table S1). Due to the high catalytic activities of GOx and HRP, the experiments were carried out with a flow rate of 10 µL/min, whereby well quantifiable absorbance values in the range of 0.5 to 1.5 a.U. (Figure 7) were obtained. However, because of the lower catalytic activity of Myo, the corresponding experiments were performed with a decreased flow rate of 5 µL/min to compensate the low activity by an increased conversion time. Based on the UV-Vis-measurements the conversion of the substrate ABTS in the microfluidic device was calculated (see Table S2). It must be noted, that the conversion in the device is rather low (up to 7.7 % for the cascade GOx-HRP, 1.1% for the cascade GOx-Myo), but can be increased upon increasing the residence time in the device as it was previously shown [14]. In the herein presented experiments, straightforward quantification of the enzymatic reaction was more important than optimization of the conversion.

To investigate the long-time functionality of the device, multiple switching cycles were performed and the catalytic conversion in the microfluidic device was monitored by continuous UV-Vis spectroscopy. The diagrams of the time-dependent absorbance visualize the dependency of the absorbance depends on the opening and closing state of the microvalves as it was intended with the type and amount of immobilized catalysts. These results show that the microvalves allow controlling the fluid pathway (Figure 7a–c, right). Thereby, due to the offline measurement of the absorbance in the flow cuvette, the absorbance changes with a timely delay and the equilibration of the measurement values (absorbance plateaus) requires some time (see Section 2.7). Apart from that, spikes of the absorbance toward higher values are detected before stable values of the absorption are obtained (see e.g., Figure 6a at a measurement time of 2 h). These spikes result from the fact, that the substrate solution remains in the HRP-containing microfluidic chamber while the empty chamber is flushed. Due to the longer residence time of the substrates at the hydrogel-HRP dots, a temporally higher conversion is achieved which is reflected by the increased absorbance. Variations of the absorbance at the absorbance plateaus result from changes of the enzyme activity which can be induced by temperature changes. Apart from that, small bubbles which can be formed by the decomposition of H_2_O_2_ can result in a decrease of the residence time of the fluid at the hydrogel array and consequently in a reduced conversion. However, despite all these effects, clearly distinguishable absorbance plateaus were achieved whereby the absorbance depended on the fluid pathway.

All in all, the long-time functionality of the microfluidic device with integrated microvalves was shown by performing the parallelized model cascade reactions with immobilized enzymes.

## 4. Conclusions

The design of a PDMS-on-glass microfluidic device with three spatially separated reaction chambers was presented. The need for microvalves as flow control elements for controlled filling and flushing of the parallel compartments was demonstrated. Consequently, electrothermally controlled hydrogel microvalves synthesized from PNiPAAm and the crosslinker BIS (diameter: 500 and 600 µm) were integrated into a valve seat in the device. Opening and closing of the valves was achieved within four and six seconds respectively and the fluid flow was thereby determined. During the development of the microfluidic system, difficulties arose from inaccurate placement of the microvalves and the formation of gas bubbles in the device. However, these problems could be solved by improvements of the experimental processes and degassing of the fluids.

Subsequently, the devices were applied to perform parallelized biocatalytic cascade reactions with the enzymes GOx and HRP as well as the protein Myo immobilized in photopatterned hydrogel dots (diameter of the dots: 350 µm, amount of enzymes: 0.13–2.3 µg) in the reaction chambers. Both the bi-enzymatic cascades GOx-HRP and GOx-Myo convert the substrates glucose and ABTS and yield [ABTS*]^+^ which was quantified by UV-Vis spectroscopy under a continuous educt flow (concentration: 5 mmol/L, flow rate: 5 or 10 µL/min). With the applied immobilization method of the enzymes, long-time activity over at least 7 h without activity loss was shown. Due to the difference in the conversion of the cascades GOx-HRP (up to 7.7 %) and for GOx-Myo (1.1 %), the opening and closing of the microvalves and thus the fluid pathway was successfully deduced from the UV-Vis spectroscopic analysis of the product solution. Thereby, long-time functionality of the microvalves in at least four switching cycles (1 h each) was achieved. Such a long opening time of hydrogel microvalves under continuous heating was to our knowledge not yet shown in the literature. Based on this proof-of-principle, the design will be used to integrate more complex parallelized multi-step cascade reactions in microfluidic devices in the future.

Still, it has to be noted that the handling of the delicate valves and accurate integration into the microfluidic chip by manual placement into the small PDMS channel is challenging and requires some expertise. The current design is rather sensitive to small deviations of the hydrogel placement which might result in non-functionality of the device. To overcome this problem, we propose the application of automatized aligning techniques as they were recently introduced for the integration of miniaturized hydrogels into microfluidic devices [26]. Additionally, despite degassing of the fluids, the formation of gas bubbles in the device upon heating could not be completely avoided. In the described application of the device for enzymatic reactions, this issue was not a drawback, as the bubbles did not fully block the channel, the enzymatic reaction was not affected, and gas bubble were released upon cooling of the device. However, for bubble-sensitive applications such as the cell cultivation [51], bubble traps could be included into the design. Alternatively, it is feasible to apply light-responsive hydrogel valves which can be switched without direct or indirect heating. In this respect, spiropiran-functionalized PNiPAAm hydrogels could come to use [30].

## Supplementary Materials

The following materials are available online at https://www.mdpi.com/2072-666X/11/2/167/s1: Figure S1: Photo of a glass slide with photopatterned hydrogel dots under daylight (**a**) and UV-light (**b**). The hydrogel dots are stained with quantum dots (left: green, top right: yellow, bottom right: non-stained); Figure S2: Photo of an array of hydrogel-valves after polymerization and washing; Figure S3: (**a**) Heat pad from polyimide foil with heat resistors R1 and R2 and (**b**) circuit diagram; Figure S4: (**a**) Photograph of the aluminum frame with transparent plexiglass window and PDMS-on-glass microfluidic device prior to alignment on one another. The location of the microvalves is marked in red. Cutouts are established in the aluminum frame to allow the fluidic connection of the device (one inlet, two bypasses, two outlets). (**b**) Photograph of the aluminum frame aligned onto the PDMS-on-glass microfluidic device; Figure S5: Microfluidic chip clamped in aluminum holder in top view (**a**) and side view (**b**). The microfluidic device is aligned on the heat pad such that the hydrogel microvalves are placed on top of the heat resistors. The location of the microvalves is marked in red; Table S1: Parameters and formulas applied for the calculation of the residence time of the substrates in the microfluidic device; Table S2: Calculation of the conversion in the microfluidic devices in dependence of the absorbance applying the formulas shown in Table S1.
