# Supplementary material for: Hydrogel Microvalves as Control Elements for Parallelized Enzymatic Cascade Reactions in Microfluidics
Source: Micromachines (Basel). 2020 Feb 5;11(2):167. doi: 10.3390/mi11020167 (PMC7074747; doi:10.3390/mi11020167)
Supplement: Supplementary file 1 [file micromachines-11-00167-s001.pdf]

micromachines

Supplementary Materials

Hydrogel Micro-Valves as Control Elements for Parallelized Enzymatic Cascade Reactions in Microfluidics

Franziska Obst, Anthony Beck, Chayan Bishayee, Philipp J. Mehner, Andreas Richter, Brigitte Voit and Dietmar Appelhans

1. Photopolymerization of the Hydrogel Arrays

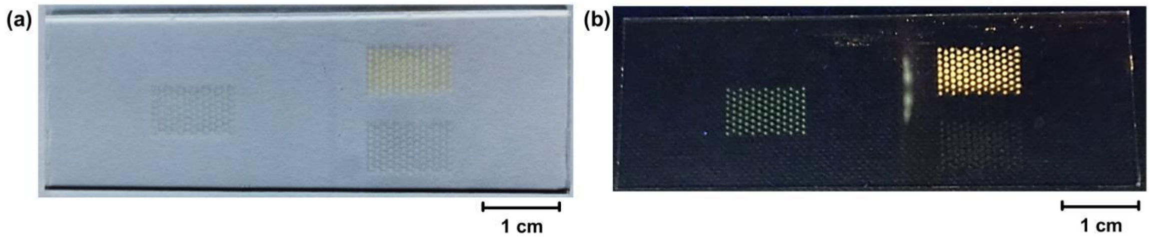

Figure S1. Photo of a glass slide with photopatterned hydrogel dots under daylight (**a**) and ultraviolet (UV)-light (**b**). The hydrogel dots are stained with quantum dots (left: green, top right: yellow, bottom right: non-stained).

2. Appearance of the Hydrogels after Polymerization and Washing

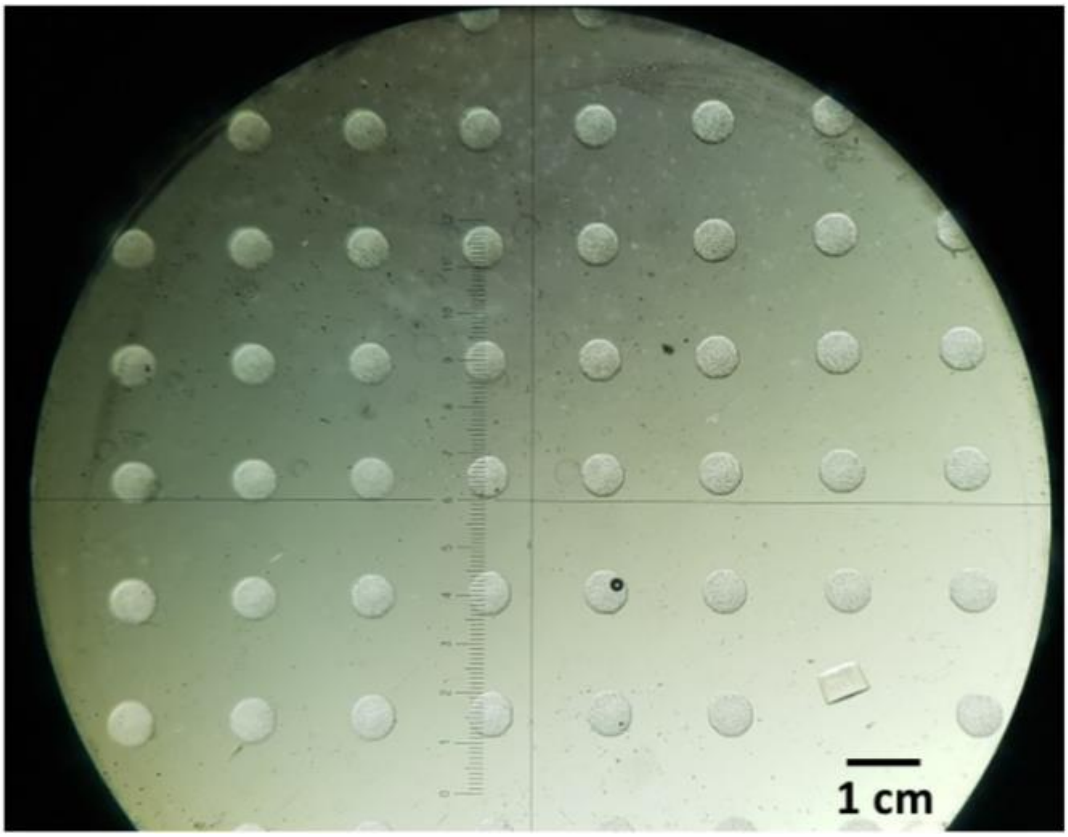

Figure S2. Photo of an array of hydrogel-valves after polymerization and washing.

3. Design of the Heat Pad

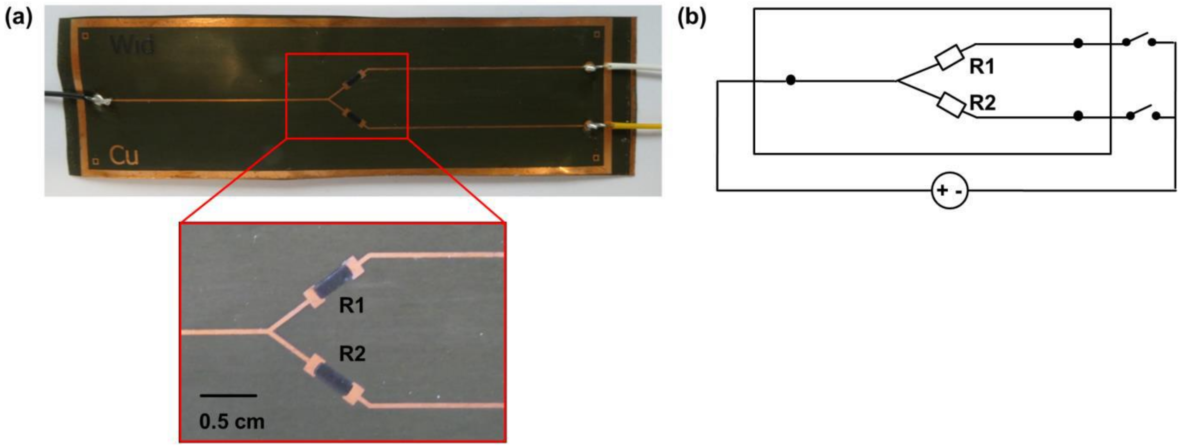

Figure S3. (**a**) Heat pad from polyimide foil with heat resistors R1 and R2 and (**b**) circuit diagram.

4. Assembly of the Microfluidic Device

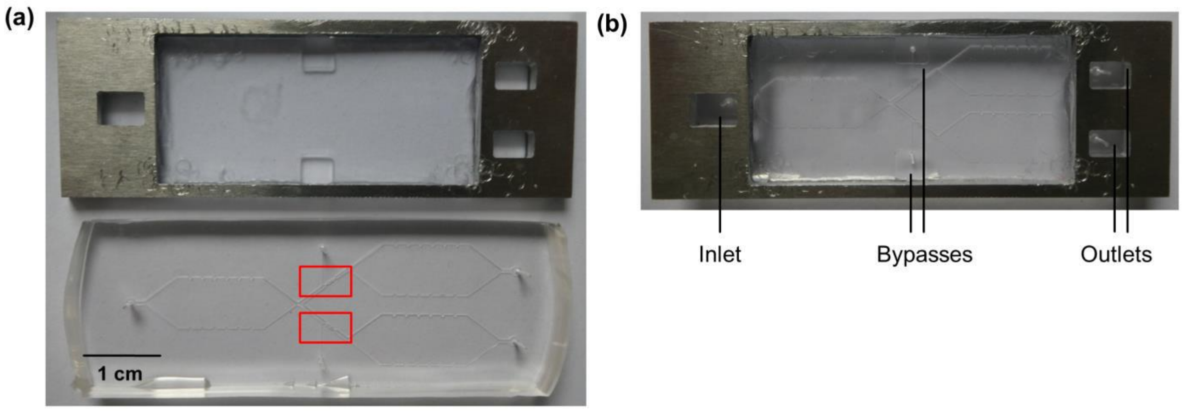

Figure S4. (**a**) Photograph of the aluminum frame with transparent plexiglass window and polydimethylsiloxane (PDMS)-on-glass microfluidic device prior to alignment on one another. The location of the microvalves is marked in red. Cutouts are established in the aluminum frame to allow the fluidic connection of the device (one inlet, two bypasses, two outlets). (**b**) Photograph of the aluminum frame aligned onto the PDMS-on-glass microfluidic device.

5. Setup for the Microfluidic Experiments

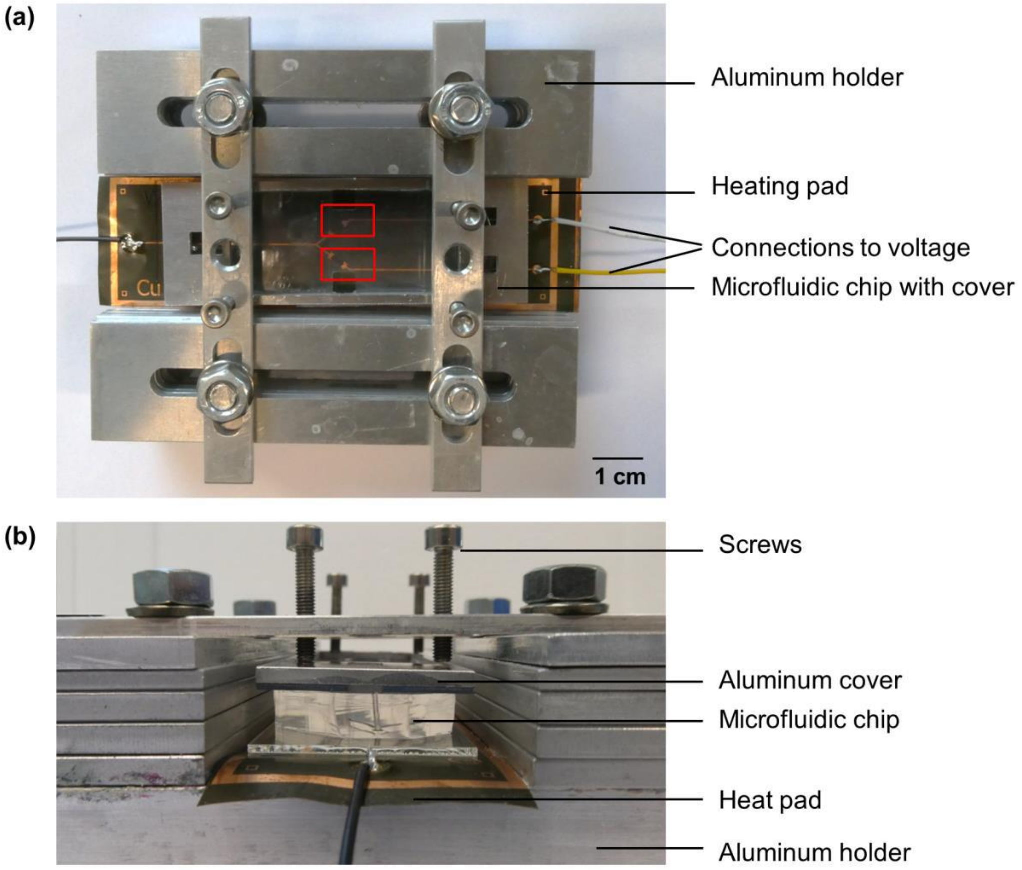

Figure S5. Microfluidic chip clamped in aluminum holder in top view (**a**) and side view (**b**). The microfluidic device is aligned on the heat pad such that the hydrogel micro-valves are placed on top of the heat resistors. The location of the micro-valves is marked in red.

6. Catalytic Activity of the Cascade Reaction GOx-Myo in Microfluidic Devices

The bi-enzymatic cascade reaction with glucose oxidase (GOx) and horseradish peroxidase (HRP) was previously carried out in microfluidic devices with both spatially separated and non-separated enzymes and the enzymatic activity in the devices was calculated. Within the present study, the bienzymatic cascade reaction with GOx and myoglobin (Myo) was established in microfluidics. For this purpose, spatially separated GOx and Myo were immobilized in photopatterned hydrogel–enzyme dots in a microfluidic device with two reaction compartments. Thereby, the reaction compartments had the same size and geometry as the compartments of the triple-chamber device. The following enzyme concentrations were adjusted in the hydrogel precursor solution: GOx: 1.17 mg/mL, Myo: 2.30 mg/mL. The catalytic activity was measured by pumping (5 μL/min) a substrate solution (glucose and 2,2′-azino-bis(3-ethylbenzothiazoline-6-sulfonic acid) diammonium salt (ABTS), 5 mmol/L) in phosphate-buffered saline (PBS) buffer (100 mmol/L, pH 6.0) through the device and continuous UV-Vis spectroscopic measurement of the product solution. The devices were each run for 1 h to reach a constant absorbance value. The experiment was repeated three times on independently fabricated microfluidic devices and the final absorbance value was 0.21 ± 0.02 a.U. Within the standard deviation, the same absorbance value was obtained for the reaction GOx–Myo in the micro-valve-controlled triple-chamber microfluidic device. Consequently, the catalytic activity of the cascade was not influenced by the integration of hydrogel microvalves into the design and electrothermal heating.

7. Calculation of the Residence Time of the Substrates and Substrate Conversion in the Microfluidic Device

Table S1. Parameters and formulas applied for the calculation of the residence time of the substrates in the microfluidic device.

**1. Geometry of the Microfluidic Device**			
Amount of hydrogel dots	ndot	98	Defined by photomask
Radius of hydrogel dots	rdot	0.175 mm	Defined by photomask (diameter: 350 µm)
Height of hydrogel dots	hdot	0.10 mm	Defined by POM mold (depth: 100 µm)
Height of microfluidic chamber	hchamber	0.15 mm	Defined by PDMS sheet (chamber depth: 150 µm)
Area of hydrogel dot array	Ahydrogel array	72.76 mm²	Defined by photomask
Volume of the microfluidic chamber	Vchamber	10.91 µL	V = Ahydrogel array × hchamber
Volume of n hydrogel dots	Vdots	0.94 µL	V = π (rdot)² × hdot
Fluid volume in microfluidic chamber	Vfluid	9.97 µL	Vfluid = Vchamber − Vdots
Residence time of fluid in microfluidic chamber (flow rate: 5 µL/min)	tR (5 µL/min)	120 s	tR = Vfluid × (flow rate)⁻¹
Residence time of fluid in microfluidic chamber (flow rate: 10 µL/min)	tR (10 µL/min)	60 s	tR = Vfluid × (flow rate)⁻¹
**2. Enzymes in the Microfluidic Chip**			
Specific enzyme activity	Aspez (GOx)<br>Aspez (HRP)<br>Aspez (Myo)	14.3 U/mg<br>76.2 U/mg<br>0.33 U/mg	Determined in ABTS assay
Enzyme concentration in hydrogel precursor	c (GOx)<br>c (HRP)<br>c (Myo)	1.17 mg/mL<br>0.40 or 0.13 mg/mL<br>2.30 mg/mL	Adjusted in device fabrication
Maximum enzyme activity in microfluidic device	Amax (GOx)<br>Amax (HRP)<br>Amax (Myo)	U	$Amax = c \times V$dots
**3. UV-Vis Measurement**			
Extinction coefficient of (ABTS*)⁺	εABTS	24.1 L/mmol cm	Determined by calibration2
Substrate concentration (glucose and ABTS)	csubs.	5 mmol/L	Applied in microfluidic experiments

Table S2. Calculation of the conversion in the microfluidic devices in dependence of the absorbance applying the formulas shown in table S1.

Experiment.	Absorbance	Conversion of ABTS (Xi) (%)
Figure 6a: V1 opened + V2 closed	0.4	2.2
Figure 6a: V2 opened + V1 closed	0.0	0.0
Figure 6b: V1 opened + V2 closed	1.4	7.7
Figure 6b: V2 opened + V1 closed	0.5	2.8
Figure 6c: V1 opened + V2 closed	1.0	5.5
Figure 6c: V2 opened + V1 closed	0.2	1.1

References

1. Obst, F.; Simon, D.; Mehner, P.J.; Neubauer, J.W.; Beck, A.; Stroyuk, O.; Richter, A.; Voit, B.; Appelhans, D. One-step photostructuring of multiple hydrogel arrays for compartmentalized enzyme reactions in microfluidic devices. *React. Chem. Eng.* **2019**, *4*, 2141–2155.
2. Simon, D.; Obst, F.; Haefner, S.; Heroldt, T.; Peiter, M.; Simon, F.; Richter, A.; Voit, B.; Appelhans, D. Hydrogel/enzyme dots as adaptable tool for non-compartmentalized multi-enzymatic reactions in microfluidic devices. *React. Chem. Eng.* **2019**, *4*, 67–77.

© 2020 by the authors. Submitted for possible open access publication under the terms and conditions of the Creative Commons Attribution (CC BY) license (http://creativecommons.org/licenses/by/4.0/).
